# Effects of arbuscular mycorrhizal dominance on taxonomic and phylogenetic beta diversity across vertical strata in a subtropical forest

**DOI:** 10.3389/fpls.2025.1675828

**Published:** 2025-09-30

**Authors:** Shuisheng Yu, Qi Wu, Jianwei Liao, Xingchen Wang, Di Ding, Rong Zheng, Libin Liu, Jianhua Chen, Julian Liu, Yunquan Wang, Mingjian Yu

**Affiliations:** ^1^ The Administration Center of Zhejiang Jiulongshan National Nature Reserve, Lishui, China; ^2^ College of Life Sciences, Zhejiang Normal University, Jinhua, China; ^3^ State Key Laboratory for Vegetation Structure, Function and Construction, MOE Key Laboratory of Biosystems Homeostasis & Protection, College of Life Sciences, Zhejiang University, Hangzhou, China

**Keywords:** beta diversity, mycorrhizal association, vertical stratification, dispersal limitation, habitat filtering, Jiulongshan subtropical forests

## Abstract

Vertical stratification in forests creates important environmental gradients that shape biodiversity patterns. While beta diversity (β-diversity) quantifies community assembly mechanisms, the relative contributions of core ecological processes, specifically biotic interactions, dispersal limitation, and habitat filtering, to taxonomic (TBD) and phylogenetic (PBD) β-diversity across vertical strata remain poorly understood. To address this knowledge gap, we quantified TBD and PBD to disentangle the relative influences of arbuscular mycorrhizal (AM) dominance (as a proxy for biotic interactions), geographic distance (as a proxy for dispersal limitation), and elevation distance (as a proxy for habitat filtering) across four vertically stratified layers (i.e., canopy, subcanopy, shrub, herb layers) in a subtropical forest. We found that species turnover is the primary component of both TBD and PBD across all strata, despite notable variation among layers. Importantly, the relative importance of these drivers on β-diversity components varied significantly across vertical strata. AM fungal dominance exerted increasing influence downward through the strata. Geographic distance became increasingly influential in the lower strata, and was the dominant driver of turnover in the herb layer. Elevation distance persistently influenced turnover components across all strata. Crucially, none of the investigated variables significantly influenced the nestedness components of either TBD or PBD. For PBD specifically, AM fungal dominance accounted for the largest proportion of variation in total PBD within the subcanopy/shrub layers, and significantly influenced specific components (turnover or nestedness) in other layers, while elevation distance exerted a strong influence on components in the subcanopy/shrub layers. However, canopy nestedness and herb-layer turnover of PBD remained decoupled from all drivers. These findings underscore the critical role of vertical stratification and highlight the importance of arbuscular mycorrhizal dominance, a key mechanism shaping community assembly, in elucidating biodiversity maintenance mechanism in structurally complex ecosystems.

## 1 Introduction

Beta diversity, which refers to the spatial variation in species composition among ecological communities, serves as a valuable lens for understanding the processes that drive the assembly and maintenance of biodiversity (Condit et al., 2002; Magurran et al., 2018; Chai et al., 2023; Xu et al., 2023). By partitioning total beta diversity into species turnover (the replacement of certain species by others) and nestedness (the pattern whereby species-poor communities form subsets of species-rich ones) (Baselga, 2010; 2012; Baselga, 2013), ecologists are able to identify the main drivers of biodiversity loss across landscapes and prioritize areas for conservation. Habitat filtering and dispersal limitation each leave distinct signatures on species turnover and nestedness: turnover dominates under high habitat heterogeneity or dispersal limitation, whereas nestedness arises from selective extinction/colonization or habitat nestedness (Socolar et al., 2016; Colville et al., 2020). Biotic interactions, such as mycorrhizal symbioses, may also modulate these patterns (Zhong et al., 2021), yet we still lack a clear understanding of how these mutualistic relationships combine with habitat filtering and dispersal constraints to shape overall beta diversity.

The structure of forest canopies governs the vertical distribution of light and water within forest ecosystems, thereby regulating carbon and water cycles and influencing forest responses to global climate change (Nakamura et al., 2017; Verheyen et al., 2024; Jucker et al., 2025). In vertically stratified stands, plants occupying different canopy layers are exposed to distinct ecological processes (Zhang et al., 2017). Overstory vegetation reduces light availability in understory layers, creating divergent resource constraints for plants occupying different vertical positions (Niinemets, 2010). In layers where resources are scarce, habitat filtering drives community assembly, whereas in strata with abundant light and space, competitive interactions lead to species exclusion and niche differentiation (Pérez-Ramos et al., 2012; Zhang et al., 2024). Dispersal processes also vary among layers because wind dispersed seeds and spores more readily colonize upper strata while animal vectors and gravity constrain propagule movement into shaded understory environments (Luo et al., 2019b). This stratification-induced divergence in deterministic processes (e.g., environmental filtering, biotic interactions) and stochastic processes (e.g., dispersal limitation) likely generates stratum-specific drivers of beta diversity. Recent empirical studies confirm that the temporal dynamics, underlying drivers, and disturbance responses of β-diversity vary vertically in (sub)tropical forests (Decocq et al., 2014; Chai et al., 2023). However, the extent to which vertical stratification shapes taxonomic and phylogenetic β-diversity, and the relative roles of these processes across strata, remains poorly understood (Chai et al., 2023).

Mycorrhizal fungi are vital members of the root-associated microbiome that trade plant-derived carbon for enhanced phosphorus and nitrogen uptake (Shi et al., 2023). Mycorrhizal symbioses influence biodiversity through habitat adaptation and plant-soil feedbacks (PSFs) (Tedersoo et al., 2020; Chaudhary et al., 2022). Arbuscular mycorrhizal (AM) associations are widespread in nutrient-rich, fast-cycling soils and frequently generate negative PSFs, as accumulating soil pathogens around dominant hosts curb their local persistence (Read and Perez-Moreno, 2003; Weber et al., 2025). In contrast, ectomycorrhizal (EcM) hosts prevail in cooler, slower-decomposing systems and often foster positive PSFs by enhancing nutrient acquisition from organic pools and reinforcing conspecific recruitment (Laliberté et al., 2015; van der Heijden et al., 2015). These dual mechanisms theoretically drive species turnover (by promoting speciation and limiting dominant species colonization) and nestedness (by selective local extinctions and fostering dominant species persistence) (Peay, 2016; Zhong et al., 2021). It has been demonstrated that with increasing latitude, AM trees tend to reduce total β-diversity and turnover, but enhance nestedness (Zhong et al., 2021). These patterns highlight the need to understand how mycorrhizae-mediated interactions shape beta diversity patterns is crucial for revealing mechanisms that maintain biodiversity, particularly in subtropical forests with mixed mycorrhizal associations.

Despite growing interest in beta diversity along horizontal gradients (Murphy et al., 2016; Luo et al., 2019a), relatively few studies have explored how mutualisms and PSFs interact with vertical habitat structure to shape community assembly. Vertically stratified subtropical forests, with their mosaic of AM- and EcM-associated trees (Mao et al., 2024), provide a natural experiment for disentangling these forces. Here, we integrate taxonomic and phylogenetic dimensions of beta diversity to investigate how mycorrhizal symbiosis, habitat filtering, and dispersal limitation jointly govern community assembly across four vertical strata in Jiulongshan forest. Specifically, we address two questions:

1. How does vertical stratification influence beta diversity components (turnover vs. nestedness) across taxonomic and phylogenetic dimensions? We hypothesized that turnover would be relatively higher in the upper layers (H1), since these strata are more susceptible to stochastic disturbance events and exhibit greater microhabitat heterogeneity, which together promote species replacement over nestedness patterns.
2. What are the stratum-specific drivers (i.e. mycorrhizal dominance, geographical distance, and elevational distance) underlying the observed diversity patterns? We hypothesized that arbuscular mycorrhizal dominance would serve as a more influential predictor in the lower strata (H2), where resource limitation enhances the importance of mycorrhiza-mediated facilitation in shaping local community.

## 2 Materials and methods

### 2.1 Study site and tree census

Our study was conducted in the Zhejiang Jiulongshan National Nature Reserve, which locates in one of the 35 priority areas for biodiversity protection in China. The reserve experiences a mid-subtropical humid monsoon climate, with a mean annual temperature of 16.2°C, an annual precipitation of 1,856 mm, and a mean annual sunshine duration of 1,925 h (Liu et al., 2024). Owing to its remote location and rugged topography, as well as the minimal level of human disturbance, the Jiulongshan reserve and its surrounding regions demonstrate exceptional biodiversity and constitute one of the most intact forest vegetation zones within the eastern mid-subtropical region of China (Liu et al., 2024).

During August 2020 to July 2023, we established 72 forest plots (20 m × 20 m, as determined by the minimum area method) distributed across representative vegetation types within the reserve. The plots were established as independent sampling units, with a minimum distance of 100 meters maintained between plots of the same forest type. A comprehensive set of environmental variables was recorded for each plot, including geographical coordinates, elevation, slope gradient, aspect orientation, and the coverage of exposed rock outcrops (Liu et al., 2025). Within each plot, all trees with a diameter at breast height (DBH) ≥ 5 cm were tagged, mapped, measured for DBH, and identified to species. Dominant tree species included *Schima superba*, *Alniphyllum fortunei* and *Castanopsis eyrei*.

We also surveyed shrub (DBH < 5cm) and herbs within eight non-adjacent 2 m × 2 m subplots per plot, nested at the northwest corner of selected quadrats. Herbs were surveyed in 1 m × 1 m subplots nested within each shrub subplot. For all shrubs and herbs, we recorded species identity, abundance, height, percent coverage, and growth status. Dominant shrub and herb species included *Eurya rubiginosa* var. *attenuata*, *Rhododendron simsii*, *Woodwardia japonica*, and *Diplopterygium glaucum*. More detailed information about plot characteristics and dominant species across vertical strata could be found in **Table 1**.

**Table 1:** Plot characteristics and dominant species across vertical strata in the Jiulongshan Nature Reserve.

Forest types	No. of plots	Range			Dominant species			
		Elevation /m	Species	Abundance	Canopy layer	Subcanopy layer	Shrub layer	Herbaceous layer
Evergreen broadleaf forests	17	500 - 1654	24 - 83	125 - 342	*Schima superba*, *Castanopsis eyrei*, and *Cyclobalanopsis glauca*	*Schima superba, Rhododendron simiarum*, and *Castanopsis eyrei*	*Syzygium buxifolium, Eurya rubiginosa* var. *attenuata*, and *Neolitsea aurata* var. *chekiangensis*	*Diplopterygium glaucum*, *Woodwardia japonica*, and *Carex* spp.
Evergreen deciduous broadleaf mixed forests	14	647 - 1579	28 - 69	91 - 304	*Schima superba, Cunninghamia lanceolata*, and *Alniphyllum fortunei*	*Schima superba, Machilus thunbergii*, and *Cunninghamia lanceolata*	*Indocalamus latifolius, Maesa japonica*, and *Machilus thunbergii*	*Selaginella moellendorffii*, *Carex* spp., and *Dryopteris setosa*
Evergreen coniferous forests	12	507 - 1643	6 - 64	74 - 329	*Pinus massoniana, Pinus taiwanensis*, and *Cunninghamia lanceolata*	*Cunninghamia lanceolata*, *Pinus massoniana, and Alniphyllum fortunei*	*Indocalamus latifolius, Eurya muricata, and Eurya rubiginosa* var. *attenuata*	*Woodwardia japonica, Dicranopteris pedata*, and *Diplopterygium glaucum*
Deciduous broadleaf forests	11	688 - 1388	24 - 83	97 - 326	*Alniphyllum fortunei, Choerospondias axillaris*, and *Tapiscia sinensis*	*Acer davidii, Prunus schneideriana, and Schima superba*	*Maesa japonica, Indocalamus latifolius, and Machilus thunbergii*	*Oplismenus undulatifolius, Carex* spp.*, and Selaginella moellendorffii*
Coniferous and broadleaf mixed forests	15	636 - 1649	32 - 74	127 - 429	*Cunninghamia lanceolata, Alniphyllum fortunei, and Pinus massoniana*	*Cunninghamia lanceolata*, *Machilus thunbergii*, and *Schima superba*	*Indocalamus latifolius*, *Eurya rubiginosa* var. *attenuata*, and *Machilus thunbergii*	*Selaginella moellendorffii*, *Carex* spp., and *Woodwardia japonica*
Bamboo forests	3	538 - 595	17 - 61	212 - 384	*Phyllostachys edulis, Cryptomeria fortunei*, and *Cunninghamia lanceolata*	*Phyllostachys edulis, Cunninghamia lanceolata*, and *Myrica rubra*	*Lindera reflexa, Cunninghamia lanceolata*, and *Camellia sinensis*	*Dicranopteris pedata, Carex* spp., and *Selaginella moellendorffii*

### 2.2 Mycorrhizal type and explanatory variables

To assess the influence of mycorrhizal association dominance on beta diversity, we assigned mycorrhizal types to each species using published databases (Brundrett and Tedersoo, 2019; Soudzilovskaia et al., 2020): arbuscular mycorrhizal (AM), ectomycorrhizal (EcM), ericoid mycorrhizal (ErM), orchid mycorrhizal (OrM), and non-mycorrhizal (NM). Species reported as dual EcM-AM associates (<0.2% of species richness) were excluded from analyses. The final dataset comprised 403 classified species: 351 AM, 27 EcM, 15 ErM, 3 OrM, and 7 NM.

Given the predominance of AM species and constraints posed by collinearity among mycorrhizal types, we focused on AM dominance (proportion of AM species abundance) as the focal mycorrhizal variable in subsequent beta diversity analyses. To evaluate the relative importance of mycorrhizal composition alongside other ecological processes (e.g., dispersal limitation and habitat filtering) in explaining beta diversity patterns, we also included geographic distance and elevational distance as explanatory variables. We employed the geosphere package to calculate geographic distances between plot centroids based on latitude and longitude (Hijmans, 2024), and vegan package calculating Euclidean distance matrices for elevational distance and AM dominance (Dixon, 2003).

### 2.3 Vertical stratification

Forests with complex vertical structure exhibit declining light availability from canopy to floor (Ishii et al., 2013), potentially driving distinct ecological processes that maintain biodiversity across strata. Based on our community surveys, we classified vegetation into three primary layers: tree, shrub, and herb. To capture finer stratification within the tree layer, we further partitioned it into canopy (trees ≥ 9.7 m height) and subcanopy (trees < 9.7 m height) sublayers using k-means clustering applied to tree height data (Hartigan & Wong, 1979). This resulted in a total of four vertically stratified layers for analysis: canopy, subcanopy, shrub, and herb.

### 2.4 Beta diversity and their components

We quantified taxonomic and phylogenetic beta diversity using pairwise Sørensen dissimilarity indices (Baselga, 2010; Leprieur et al., 2012). To elucidate potential differences in the ecological processes driving distinct components of beta diversity, we partitioned the Sørensen dissimilarity into two additive components: turnover and nestedness. The turnover component reflects species (or phylogenetic branch) replacement independent of differences in species richness (or phylogenetic diversity) between sites (Baselga, 2010; Leprieur et al., 2012). The nestedness component quantifies the contribution of richness differences, where species-poor assemblages represent subsets of species-rich assemblages (Baselga, 2010; Leprieur et al., 2012). To assess the relative importance of turnover versus nestedness in structuring overall beta diversity, we further calculated multiple-site Sørensen dissimilarity indices and their respective turnover and nestedness components (Zhao et al., 2021).

We quantified abundance-weighted taxonomic beta diversity (*β*_SOR_) and their components (turnover, *β*_SIM_; nestness, *β*_SNE_) as follows (Equations 1–3) (Baselga, 2013):


(1) $${\text{β}}_{\text{SOR}}=\frac{\text{B}+\text{C}}{2\text{A}+\text{B}+\text{C}}$$

(2) $${\text{β}}_{\text{SIM}}=\frac{\text{min}(\text{B},\text{C})}{\text{A}+\text{min}(\text{B},\text{C})}$$

(3) $${\text{β}}_{\text{SNE}}=\frac{\left|\text{B}-\text{C}\right|}{2A+\text{B}+\text{C}}\times \frac{\text{A}}{\text{A}+\text{min}(\text{B},\text{C})}$$

where *A* is the number of individuals shared between two communities, and *B* and *C* represent the number of individuals unique to each community, respectively (Baselga, 2010).

A regional mega-tree (GBOTB.extended.tre;Jin and Qian, 2022) was pruned to include only species recorded in our study. Species taxonomy was standardized following World Plants (https://www.worldplants.de) and the APG IV system (The Angiosperm Phylogeny Group, 2016). Analogous to the taxonomic beta diversity calculations, we computed the phylogenetic beta diversity (*β*_PHY.SOR_) and its turnover (*β*_PHY.SIM_) and nestedness (*β*_PHY.SNE_) components based on our phylogenetic tree. This was achieved by replacing the following branch-length equivalents into Equations 1-3 (Leprieur et al., 2012):


(4) $$\text{A}=\sum_{{\text{T}}_{\text{j}}\cap {\text{T}}_{\text{k}}}{\text{w}}_{\text{t}}$$

(5) $$\text{B}=\sum_{{\text{T}}_{\text{j}}\cup {\text{T}}_{\text{k}}}{\text{w}}_{\text{t}}-\sum_{{\text{T}}_{\text{k}}}{\text{w}}_{\text{t}}$$

(6) $$\text{C}=\sum_{{\text{T}}_{\text{j}}\cup {\text{T}}_{\text{k}}}{\text{w}}_{\text{t}}-\sum_{{\text{T}}_{\text{j}}}{\text{w}}_{\text{t}}$$

Here, *T_j_* and *T_k_* represent the subtrees of a rooted regional phylogenetic tree *T* for communities *j* and *k*, respectively. Each branch *t* in the tree *T* has a length *w_t_*. A is the sum of branch lengths shared between communities *j* and *k* (Equation 4), *B* and *C* is the sum of branch lengths unique to community *j* and community *k*, respectively (Equations 5, 6). The U.Taxonstand package was employed to standardize taxonomy (Zhang and Qian, 2023), while the U.PhyloMaker package was applied to construct the phylogenetic tree (Jin and Qian, 2022; 2023). Both taxonomic and phylogenetic beta diversity were calculated by using betapart package (Baselga and Orme, 2012).

### 2.5 Statistical analysis

To test for significant differences in total beta diversity and components (turnover and nestness) among the four vertical strata (canopy, subcanopy, shrub, herb), we used pairwise Wilcoxon rank-sum tests with Bonferroni correction for multiple comparisons. To analyze whether the drivers of beta diversity varied across vertical strata, we employed a two-step approach for each stratum separately. First, we used Mantel tests (Spearman’s rank correlation, 9999 permutations) to evaluate the associations between pairwise community dissimilarity matrices (each beta diversity component) and matrices of geographic distance, elevational distance, and AM dominance (Fang et al., 2024). Second, for beta diversity components showing significant Mantel correlations (*P* < 0.05) with any of the explanatory distance matrices, we performed Multiple Regression on Distance Matrices (MRM) to quantify the relative contributions of the significant drivers (Guimarães et al., 2021) by using ecodist package (Goslee and Urban, 2007). Significance of MRM coefficients was assessed using 9999 permutations. All analyses were conducted in R version 4.3.2 (https://www.r-project.org/).

## 3 Results

### 3.1 Stratified patterns of taxonomic and phylogenetic beta diversity components

**Figure 1:**
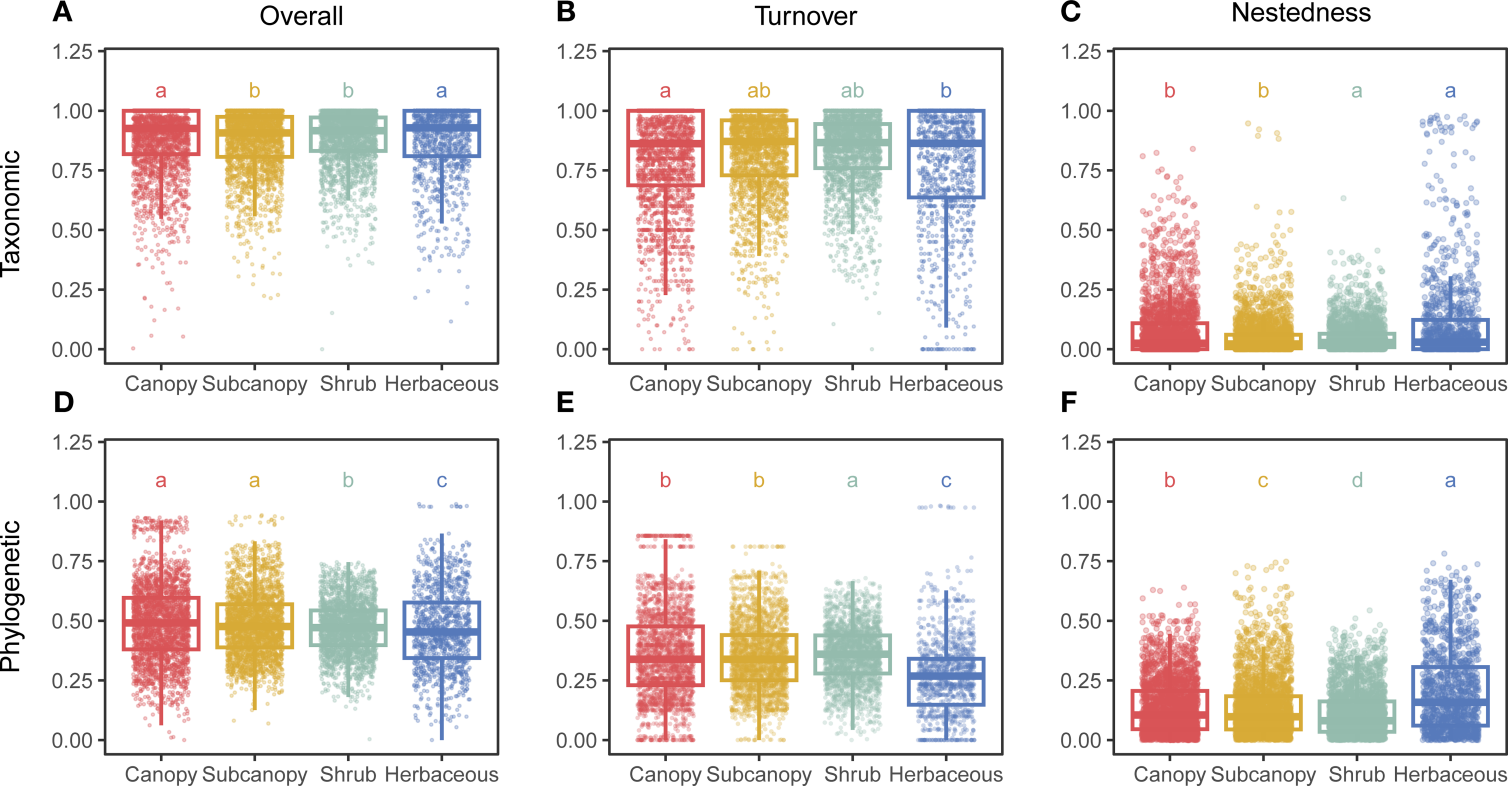
Vertical stratification of taxonomic and phylogenetic beta diversity components. Bar plots show differences in **(A–C)** taxonomic beta diversity and its turnover and nestedness components, and **(D–F)** phylogenetic beta diversity and its turnover and nestedness components across four vertical strata (Canopy, Subcanopy, Shrub, Herb). Different lowercase letters indicate significant differences among strata based on pairwise Wilcoxon rank-sum tests with Bonferroni correction (*p* < 0.05).

Our analysis revealed distinct vertical stratification patterns in both taxonomic beta diversity (TBD) and phylogenetic beta diversity (PBD) components. Significantly higher TBD was observed in the canopy and herbaceous layers compared to the subcanopy and shrub layers (**Figure 1**). In contrast, PBD was significantly higher in the canopy and subcanopy layers than in the shrub and herbaceous layers (**Figure 1**).

Both TBD and PBD were strongly dominated by the turnover component (**Supplementary Figure S1**). Taxonomic turnover was highest in the canopy layer, while phylogenetic turnover peaked in the shrub layer, Conversely, the nestedness component of TBD was elevated in the shrub and herbaceous layers (**Figure 1**). For PBD, the nestedness component decreased progressively from the herbaceous layer to the canopy, subcanopy, and shrub layers, with significant differences between all adjacent strata (**Figure 1**).

### 3.2 Stratum-specific biotic and abiotic drivers of beta diversity components

**Figure 2:**
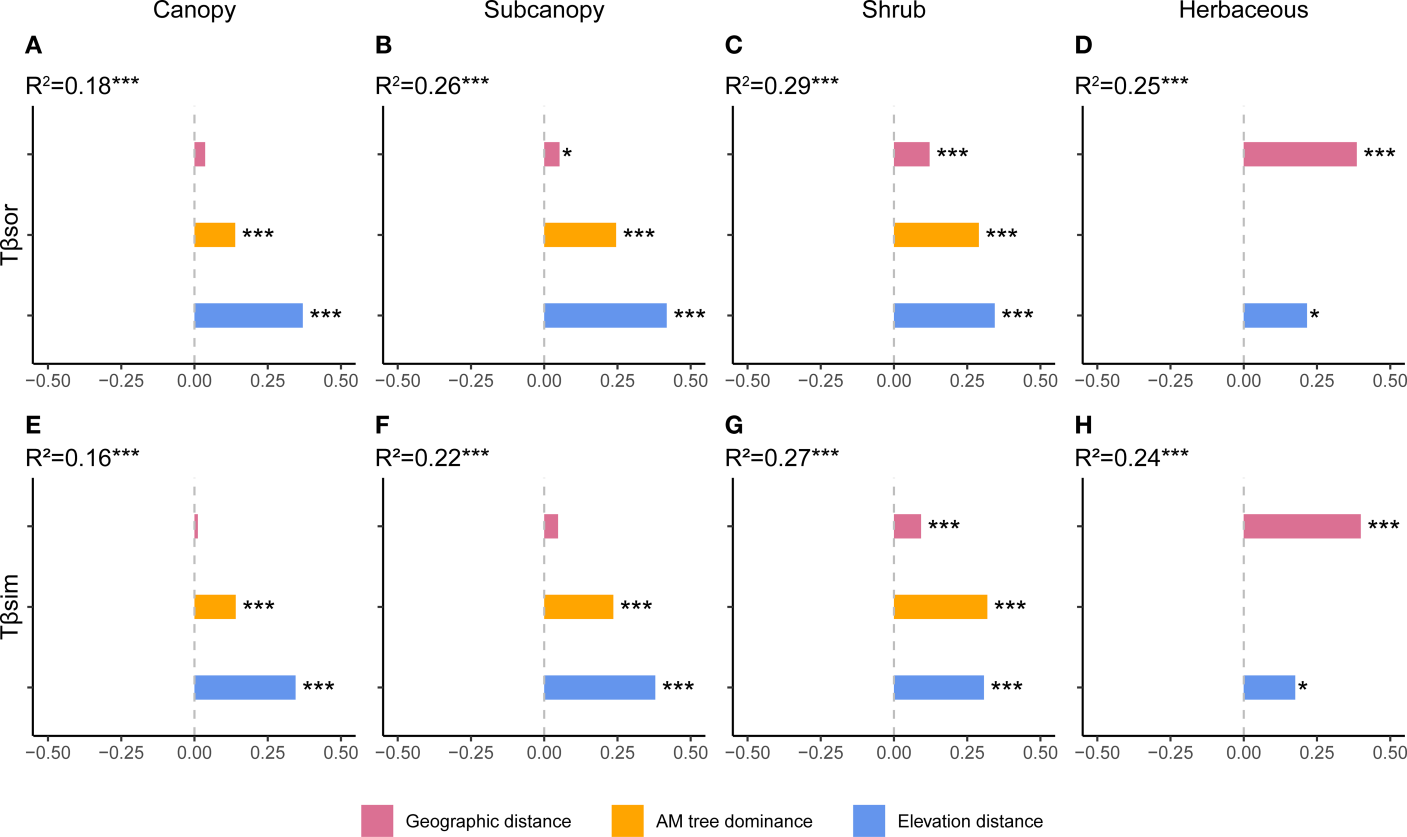
Drivers of taxonomic beta diversity components across vertical strata. Results of multiple regression on distance matrices (MRM) showing the proportion of variance explained by arbuscular mycorrhizal (AM) dominance, geographic distance, and elevational distance for **(A–D)** total taxonomic beta diversity and **(E–H)** its turnover component in each stratum. The nestedness component is not shown as no significant relationships were found for any driver in any stratum. *p < 0.05, **p < 0.01, ***p < 0.001.

Total TBD and its turnover component displayed similar driving factors (**Figure 2**, **Supplementary Figure S2**). The influence of AM fungal dominance increased progressively from the canopy through the subcanopy to the shrub layer but was non-significant in the herb layer (**Figure 2**). Notably, AM dominance exhibited its highest relative importance in the turnover component of the shrub layer (**Figure 2G**). Geographic distance showed increasing explanatory power from upper to lower strata, becoming the most influential variable in the herb layer (**Figures 2D, H**). Crucially, while geographic distance did not significantly affect total TBD in the canopy, it significantly explained turnover in understory strata (shrub and herb layers; **Figure 2**). Elevation distance remained significant across all layers (**Figure 2**). Crucially, none of the three drivers significantly influenced the nestedness component of TBD (**Figure 2**).

**Figure 3:**
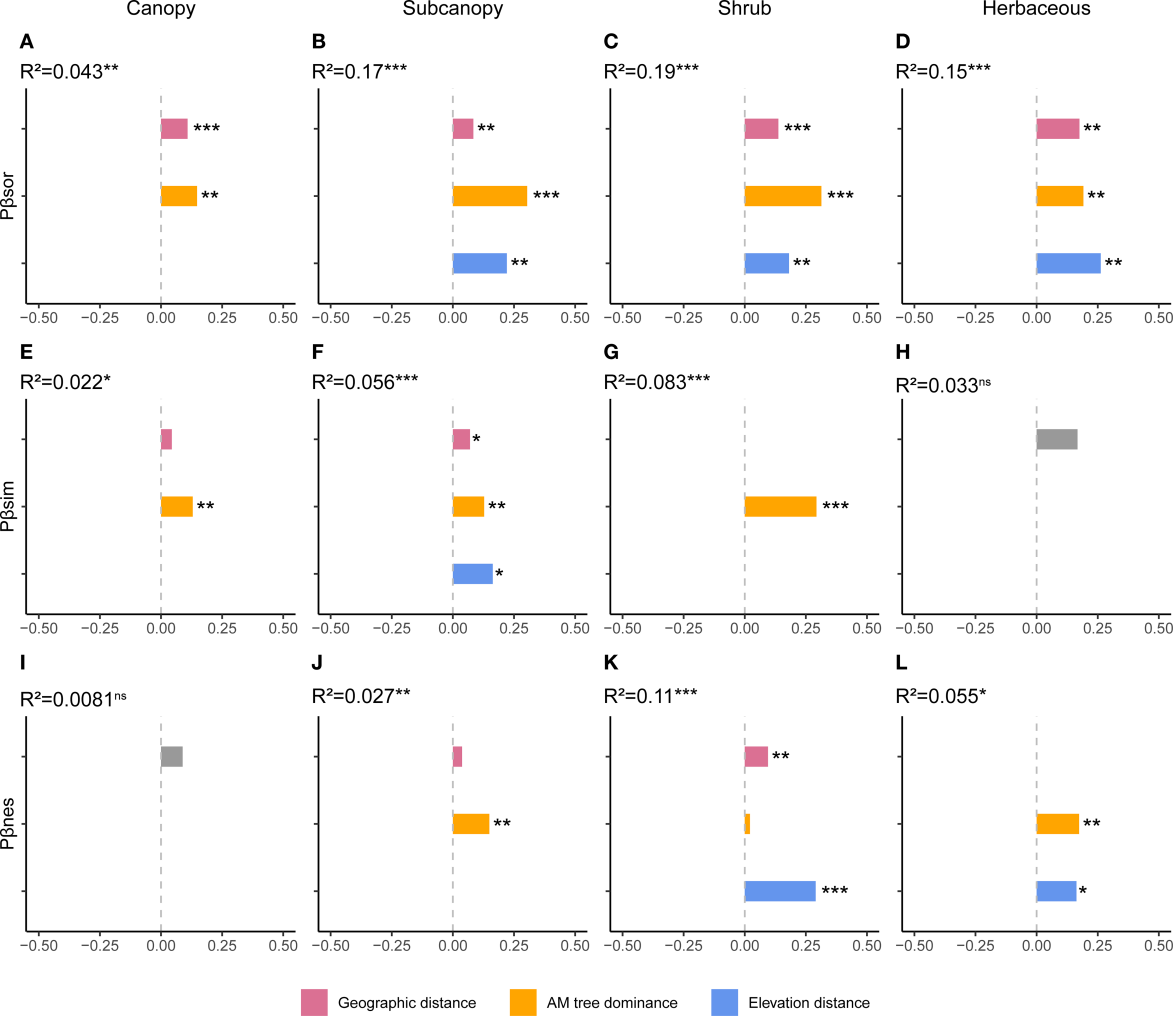
Drivers of phylogenetic beta diversity components across vertical strata. Results of multiple regression on distance matrices (MRM) showing the proportion of variance explained by arbuscular mycorrhizal (AM) dominance, geographic distance, and elevational distance for **(A–D)** total phylogenetic beta diversity, **(E–H)** its turnover component, and **(I–L)** its nestedness component in each stratum. *p < 0.05, **p < 0.01, ***p < 0.001.

Total PBD and its component displayed complex strata-dependent drivers (**Figure 3**, **Supplementary Figure S3**). For total PBD, AM dominance accounted for the greatest proportion of variation in the subcanopy and shrub layers (**Figures 3B, C**). Geographic distance was significant across all strata, while elevation distance was non-significant only in the canopy but significant in all other strata (**Figures 3A-D**). Regarding PBD components, AM fungal dominance exhibited peak explanatory power for turnover in the canopy and shrub layers, and for nestedness in the subcanopy and herb layers (**Figure 3**). Elevation distance was the most important explanatory variable for both PBD components in the subcanopy and shrub layers (**Figure 3**). All examined variables exhibited no significant effects on the nestedness component in the canopy layer or on the turnover component in the herb layer (**Figures 3J, H**).

## 4 Discussion

### 4.1 Stratification of taxonomic and phylogenetic beta diversity components

When examining beta diversity across spatial gradients, specifying the conceptual type of beta diversity is crucial, as different types yield distinct patterns and interpretations (Anderson et al., 2011). Our analysis of taxonomic beta diversity (TBD), phylogenetic beta diversity (PBD), and their respective turnover and nestedness components revealed pronounced vertical stratification within the forest. TBD peaked in the canopy and herbaceous layers (**Figure 1**), suggesting these strata exhibit the greatest species compositional variation along the vertical gradient, likely driven by distinct suites of species adapted to contrasting conditions at the forest top and bottom (Murphy et al., 2016; Deng et al., 2025). Conversely, heightened PBD in the canopy and subcanopy layers indicates stronger habitat filtering acting on evolutionary lineages adapted to distinct vertical microclimates (Shen et al., 2022). These findings demonstrate that different forest strata contribute distinctively to overall biodiversity, underscoring the necessity of considering vertical stratification in conservation strategies aiming to preserve both taxonomic and phylogenetic diversity (Oliveira and Scheffers, 2019).

Both TBD and PBD were overwhelmingly dominated by the turnover component (**Supplementary Figure S1**). This dominance of turnover holds significant ecological implications for the SLOSS (Single Large or Several Small) debate in reserve design (Gutiérrez-Cánovas et al., 2013). When nestedness predominates, protecting species-rich communities may suffice for conservation (Deane et al., 2020). Conversely, the pervasive turnover dominance observed across all vertical strata at Jiulongshan necessitates the protection of multiple distinct communities to maximize biodiversity conservation (Socolar et al., 2016). The consistently low contribution of the nestedness component was anticipated. While clonal propagation (e.g., in understory bamboos like *Indocalamus latifolius* or *I. tessellatus*) could generate localized nestedness patterns near parent plants (Ulrich et al., 2009), its influence diminishes rapidly with distance and is unlikely to structure beta diversity significantly at our sampling scale.

The beta diversity components also reflect distinct patterns along the vertical gradient. Taxonomic turnover peaked in the canopy layer, whereas phylogenetic turnover was highest in the shrub layer (**Figure 1**). Forest canopy, as the primary interface between the forest and atmosphere, is highly exposed to disturbances that can generate stochastic colonization events, and its structural heterogeneity creates pronounced microclimatic gradients (Nakamura et al., 2017). Consistent with H1, canopny layer may foster higher species replacement rates compared to lower strata. The shrub layer, constrained by light limitation from above and influenced by resource availability from below (Onoda et al., 2014; Matsuo et al., 2021), exhibits finely partitioned niches that support phylogenetically divergent lineages. Notably, the nestedness component of TBD was elevated in the shrub and herbaceous layers, whereas phylogenetic nestedness reached its minimum in the shrub stratum (**Figure 1**). This pattern suggests that localized disturbances near the forest floor (e.g., branch fall) may drive selective species loss. Such deterministic extinction processes are known to increase nestedness in understory communities (Qian, 2009; Platt et al., 2015). Collectively, H1 is supported for TBD turnover rather than PBD tunover in the canopy layer, while nestedness showed more complex and layer-specific patterns.

### 4.2 Vertical variation in drivers of forest beta diversity components

Our study assessed how arbuscular mycorrhizal (AM) dominance, geographic distance, and elevational distance shape taxonomic (TBD) and phylogenetic (PBD) beta diversity across strata, reflecting deterministic and stochastic processes driving community dissimilarity. We argue that these factors reflect central ecological processes driving beta diversity. Mycorrhizal fungi form mutualistic symbioses with plant roots, enhancing host access to nutrients like phosphorus and nitrogen (Shi et al., 2023; Eagar et al., 2025). Conversely, dispersal limitation predicts increasing beta diversity with geographic distance, as restricted movement reduces similarity between distant communities (Bell, 2001; Green and Ostling, 2003). Furthermore, elevational differences create environmental filters (e.g., temperature, precipitation gradients) that select adapted species from regional pools (Loreau, 2000). While most previous studies emphasize abiotic drivers like geographic and elevational distance (Hu et al., 2022; Fares et al., 2025), our work highlights the significant role of biotic mutualisms indicated by AM dominance.

Vertical stratification fundamentally altered the influence of key biotic and abiotic drivers on beta diversity components. For total TBD and its turnover component, the explanatory power of AM fungal dominance increased from the canopy to the shrub layer, becoming dominant in the shrub layer for turnover as our H2 (**Figure 2**). This aligns with the shrub layer’s exposure to dual resource constraints from both upper and lower layers, supporting the stress-gradient hypothesis which predicts increased facilitation under resource-limited conditions (Zhang et al., 2024; Beauchamp et al., 2025). Geographic distance showed increasing explanatory power from upper to lower strata, indicating stronger dispersal limitation in the understory layer, particularly influencing species turnover (**Figure 2**). This likely stems from the lower stature and weaker long-distance dispersal potential of understory plants (Torres et al., 2022), making their composition more directly susceptible to geographic isolation. Elevation distance remained significant across all strata (**Figure 2**), consistent with the established importance of habitat filtering (Peay, 2016; Zellweger et al., 2017). Strikingly, none of these drivers explained the nestedness component of TBD in any stratum (**Figure 2**). This suggests unmeasured processes, such as canopy structural heterogeneity (Zhou et al., 2020), may cause poorer sites to represent subsets of species from richer sites, generating nestedness patterns. Overall, in terms of TBD, deterministic processes dominate in resource−limited strata (e.g., subcanopy layer, partially supported H2), while stochastic processes prevail in more isolated (herb layer) or disturbance−prone (canopy layer) layers.

Patterns in phylogenetic beta diversity (PBD) were more complex, differing across strata and between total PBD and its components. For total PBD, AM dominance explained the highest proportion of variation in the subcanopy and shrub layers (**Figure 3**). Mechanistically, negative PSFs in AM trees may drive abundance-weighted turnover by enhancing replacement with habitat-specialized heterospecifics, particularly rare species (Bennett et al., 2017; Liu and He, 2019). Geographic and elevation distances remained significant predictors in most strata (**Figure 3**), indicating conserved niche evolution where closely related species experience similar dispersal limitation and habitat filtering (Carvajal-Endara et al., 2017). For PBD components, AM dominance primarily influenced turnover in canopy and shrub layers but governed nestedness in subcanopy and herb layers (**Figure 3**), demonstrating how identical processes can drive distinct assembly outcomes across spatial gradient (He et al., 2024). Elevation distance emerged as the strongest predictor in middle strata (subcanopy and shrub layers; **Figure 3**), reflecting phylogenetically conserved habitat filtering under their compounded vertical stresses (Emerson and Gillespie, 2008). In summary, PBD patterns indicate that deterministic and stochastic processes interact in varying combinations across strata, producing more complex vertical assembly dynamics than in TBD.

Several limitations warrant consideration. First, non-significant effects of our focal drivers (AM dominance, geographic/elevation distance) on some dimensions of beta diversity (e.g., turnover and nestedness components of PBD in certain strata) suggest incomplete capture of key ecological processes structuring beta diversity. Second, while positive plant-soil feedbacks (PSFs) in EcM trees mediated by pathogen-protective root mantles may promote conspecific success and potentially drive nestedness through competitive exclusion (Peay, 2016; Bennett et al., 2017), co-linearity between AM and EcM dominance precluded validation of this pathway. Future investigations should examine non-AM mycorrhizal systems and incorporate root functional traits to refine these mechanistic insights.

## 5 Conclusions

This study analyzed the effects of arbuscular mycorrhizal (AM) dominance, geographic distance, and elevational distance on the components (turnover and nestedness) of taxonomic and phylogenetic beta diversity across vertical strata. We found distinct vertical distribution patterns for both diversity dimensions, but turnover overwhelmingly dominated beta diversity in all cases. The drivers of beta diversity also varied considerably with stratum height. Patterns for total taxonomic diversity and its turnover component were similar, while drivers for the nestedness component of taxonomic diversity remained unclear. In contrast, patterns for total phylogenetic diversity, its turnover, and its nestedness components differed more substantially across strata. Importantly, AM dominance was a key driver in specific strata and diversity dimensions, highlighting the importance of facilitation by mycorrhizal symbiosis in shaping biodiversity. Our study demonstrates that vertical stratification fundamentally modulates the drivers of beta diversity in subtropical forests. This work establishes that biodiversity assembly mechanisms are distinctly stratified within forests, emphasizing that conservation requires integrated consideration of the entire vertical context. In addition, we recommend that management practices prioritize the protection of forest vertical structural complexity, while incorporating the mycorrhizal types of plants, to develop more scientifically robust and effective biodiversity conservation strategies.

## Data Availability

The original contributions presented in the study are included in the article/**Supplementary Material**. Further inquiries can be directed to the corresponding authors.

## Supplementary material

Supplementary Table 1 Mantel test results for both taxonomic and phylogenetic beta diversity across vertical strata in Jiulongshan Nature Reserve.

Supplementary Table 2 The relative importance of variables influencing both taxonomic and phylogenetic beta diversity based on MRM models in Jiulongshan Nature Reserve.

Supplementary Figure 1 Relative contribution of turnover to total beta diversity across vertical strata. Proportion of **(A)** taxonomic beta diversity and **(B)** phylogenetic beta diversity explained by the turnover component in each stratum.

Supplementary Figure 2 Correlations between drivers and taxonomic beta diversity components across vertical strata. Mantel test results showing relationships of arbuscular mycorrhizal (AM) dominance, geographic distance, and elevational distance with: **(A-D)** Total taxonomic beta diversity, **(E-H)** Turnover component, **(I-L)** Nestedness component. **p* < 0.05, ***p* < 0.01, ****p* < 0.001.

Supplementary Figure 3 Correlations between drivers and phylogenetic beta diversity components across vertical strata. Mantel test results showing relationships of arbuscular mycorrhizal (AM) dominance, geographic distance, and elevational distance with: **(A-D)** Total phylogenetic beta diversity, **(E-H)** Turnover component, **(I-L)** Nestedness component. **p* < 0.05, ***p* < 0.01, ****p* < 0.001.
