# Supplementary material for: Effects of arbuscular mycorrhizal dominance on taxonomic and phylogenetic beta diversity across vertical strata in a subtropical forest
Source: Front Plant Sci. 2025 Sep 30;16:1675828. doi: 10.3389/fpls.2025.1675828 (PMC12518276; doi:10.3389/fpls.2025.1675828)

Effects of arbuscular mycorrhizal dominance on taxonomic and phylogenetic beta diversity across vertical strata in a subtropical forest

Shuisheng Yu[1†], Qi Wu [2†], Jianwei Liao[1], Xingchen Wang[2], Di Ding[2], Rong Zheng[2], Libin Liu[1,2,3], Jianhua Chen[2], Julian Liu[1*], Yunquan Wang[2,3*], Mingjian Yu[2,3]

[1]The Administration Center of Zhejiang Jiulongshan National Nature Reserve, Lishui 323300, China

[2]College of Life Sciences, Zhejiang Normal University, Jinhua 321004, China

[3]State Key Laboratory for Vegetation Structure, Function and Construction, MOE Key Laboratory of Biosystems Homeostasis & Protection and College of Life Sciences, College of Life Sciences, Zhejiang University, Hangzhou 310058, China

Supplementary Materials:

Table S1 Mantel test results for both taxonomic and phylogenetic beta diversity across vertical strata in Jiulongshan Nature Reserve.

Vertical strata	Variables	Taxonomic β-diversity	Taxonomic turnover	Taxonomic nestedness	Phylogenetic β-diversity	Phylogenetic turnover	Phylogenetic nestedness
Canopy layer	Elevation distance	0.40	0.37		-	-	-
Canopy layer	AM plant dominance	0.19	0.18	-	0.16	0.13	-
Canopy layer	Geographic distance	0.11	0.08	-	0.12	0.06	0.09
Subcanopy layer	Elevation distance	0.45	0.41	-	0.26	0.19	-
Subcanopy layer	AM plant dominance	0.28	0.27	-	0.32	0.14	0.15
Subcanopy layer	Geographic distance	0.14	0.13	-	0.14	0.11	0.05
Shrub layer	Elevation distance	0.44	0.40	-	0.28	-	0.31
Shrub layer	AM plant dominance	0.38	0.40	-	0.37	0.27	0.10
Shrub layer	Geographic distance	0.20	0.16	-	0.19	-	0.15
Herbaceous layer	Elevation distance	0.31	0.27	-	0.30	-	0.16
Herbaceous layer	AM plant dominance	-	-	-	0.17	-	0.16
Herbaceous layer	Geographic distance	0.44	0.45	-	0.24	0.17	-

Note: The symbol “-” indicates that the result is not statistically significant.

Table S2 The relative importance of variables influencing both taxonomic and phylogenetic beta diversity based on MRM models in Jiulongshan Nature Reserve.

MRM models	Canopy layer	Subcanopy layer	Shrub layer	Herbaceous layer
Taxonomic β-diversity				
Ele+Geo+AM	0.18	0.26	0.29	0.25
Ele+Geo	0.16	0.20	0.21	0.24
Ele+AM	0.18	0.26	0.27	0.11
Geo+AM	0.05	0.10	0.18	0.20
Ele	0.16	0.20	0.19	0.10
Geo	0.01	0.02	0.04	0.20
AM	0.03	0.08	0.15	0.01
Phylogenetic β-diversity				
Ele+Geo+AM	0.04	0.17	0.19	0.15
Ele+Geo	0.02	0.08	0.10	0.11
Ele+AM	0.03	0.16	0.17	0.12
Geo+AM	0.04	0.12	0.16	0.09
Ele	0.01	0.07	0.08	0.09
Geo	0.02	0.02	0.04	0.06
AM	0.03	0.11	0.13	0.03

Note: Ele denotes the elevation distance, Geo denotes the geographical distance, and AM denotes the arbuscular mycorrhizal plant dominance.

Figure S1 Relative contribution of turnover to total beta diversity across vertical strata. Proportion of (a) taxonomic beta diversity and (b) phylogenetic beta diversity explained by the turnover component in each stratum.

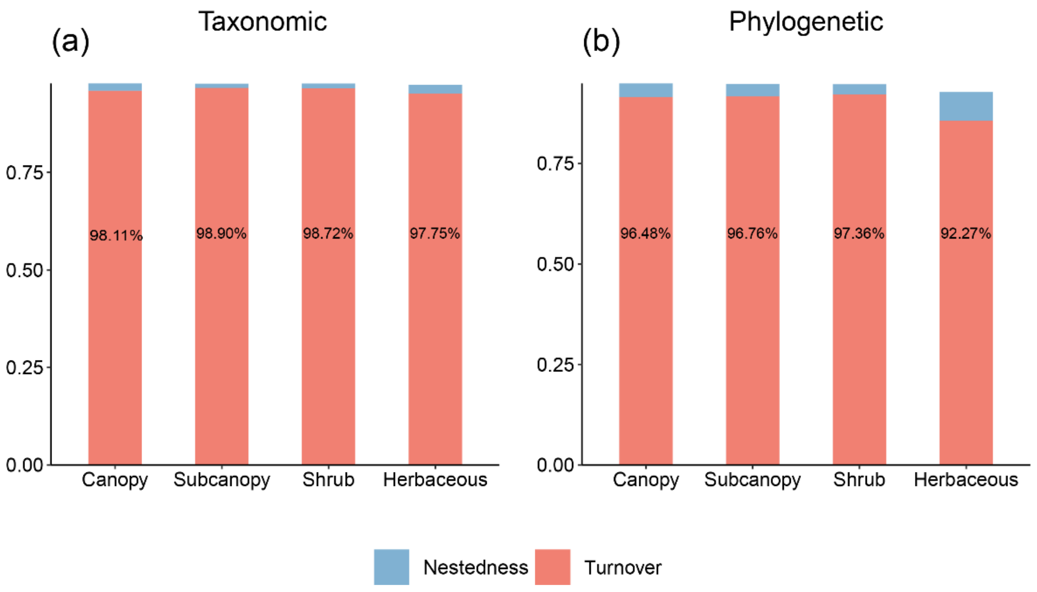

Figure S2 Correlations between drivers and taxonomic beta diversity components across vertical strata. Mantel test results showing relationships of arbuscular mycorrhizal (AM) dominance, geographic distance, and elevational distance with: (a-d) Total taxonomic beta diversity, (e-h) Turnover component, (i-l) Nestedness component. **p* < 0.05, ***p* < 0.01, ****p* < 0.001.

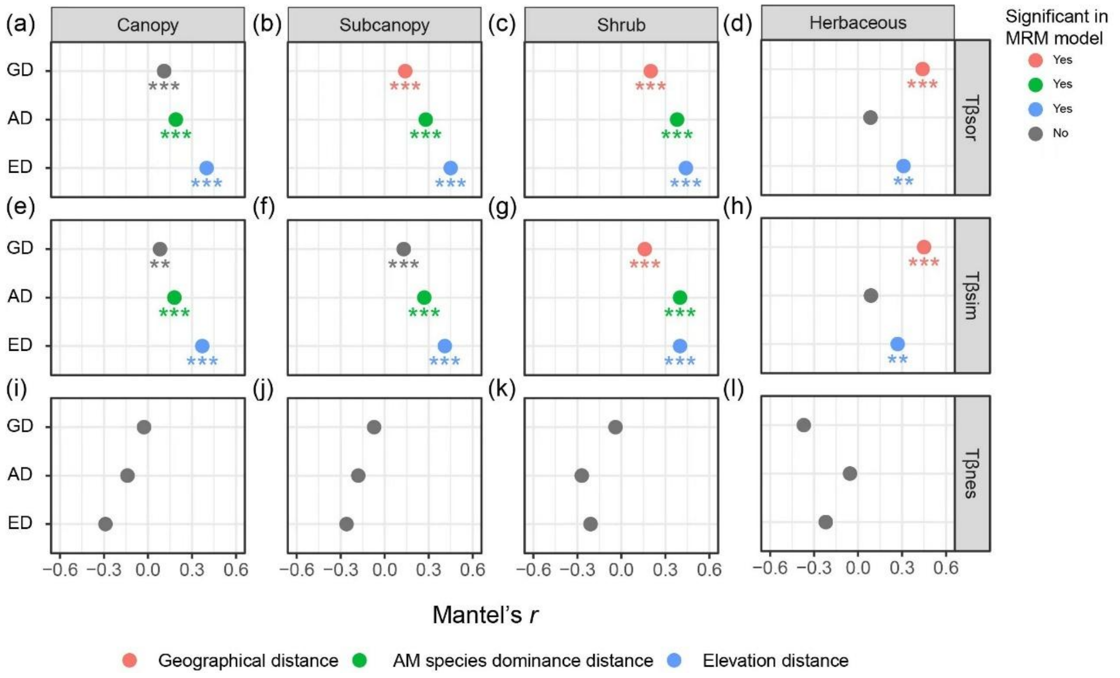

Figure S3 Correlations between drivers and phylogenetic beta diversity components across vertical strata. Mantel test results showing relationships of arbuscular mycorrhizal (AM) dominance, geographic distance, and elevational distance with: (a-d) Total phylogenetic beta diversity, (e-h) Turnover component, (i-l) Nestedness component. *$p < 0.05$, **$p < 0.01$, ***$p < 0.001$.

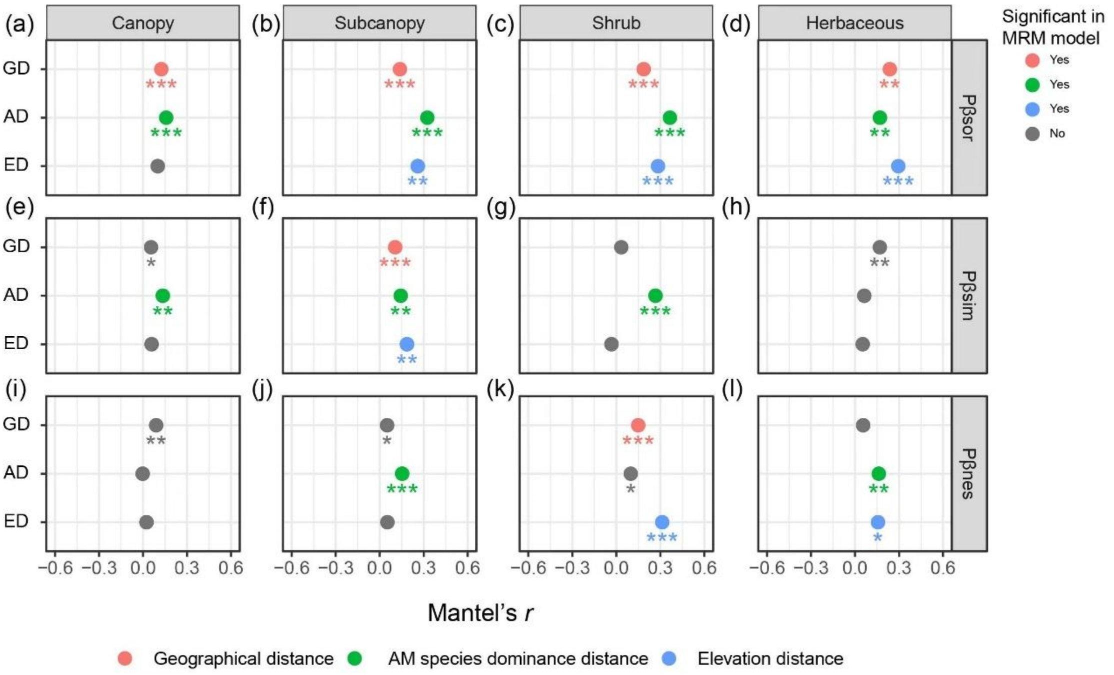

Supplement: Supplementary Table 1 — Mantel test results for both taxonomic and phylogenetic beta diversity across vertical strata in Jiulongshan Nature Reserve. [file DataSheet1.pdf]
